# Sex and the single embryo: early deveopment in the Mediterranean fruit fly, *Ceratitis capitata*

**DOI:** 10.1186/1471-213X-10-12

**Published:** 2010-01-26

**Authors:** Paolo Gabrieli, Andrea Falaguerra, Paolo Siciliano, Ludvik M Gomulski, Francesca Scolari, Antigone Zacharopoulou, Gerald Franz, Anna R Malacrida, Giuliano Gasperi

**Affiliations:** 1 Department of Animal Biology, University of Pavia, Piazza Botta 9, 27100 Pavia, Italy; 2 Entomology Unit, FAO/IAEA Agriculture and Biotechnology Laboratory, Joint FAO/IAEA Programme, International Atomic Energy Agency, Vienna, Austria; 3 Division of Genetics, Cell and Developmental Biology, Department of Biology, University of Patras, 26500 Patras, Greece

## Abstract

**Background:**

In embryos the maternal-to-zygotic transition (MTZ) integrates post-transcriptional regulation of maternal transcripts with transcriptional activation of the zygotic genome. Although the molecular mechanisms underlying this event are being clarified in *Drosophila melanogaster*, little is know about the embryogenic processes in other insect species. The recent publication of expressed sequence tags (ESTs) from embryos of the global pest species *Ceratitis capitata *(medfly) has enabled the investigation of embryogenesis in this species and has allowed a comparison of the embryogenic processes in these two related dipteran species, *C. capitata *and *D. melanogaster*, that shared a common ancestor 80-100 mya.

**Results:**

Using a novel PCR-based sexing method, which takes advantage of a putative LTR retrotransposon MITE insertion on the medfly Y chromosome, the transcriptomes of individual early male and female embryos were analysed using RT-PCR. This study is focused on two crucial aspects of the onset of embryonic development: sex determination and cellular blastoderm formation. Together with the three known medfly genes (*Cctransformer*, *Cctransformer2 *and *Ccdoublesex*), the expression patterns of other medfly genes that are similar to the *D. melanogaster *sex-determination genes (*sisterlessA, groucho, deadpan, Sex-lethal, female lethal d, sans fille *and *intersex*) and four cellular blastoderm formation genes (*Rho1, spaghetti squash, slow-as-molasses *and *serendipity-α*) were analyzed, allowing us to sketch a preliminary outline of the embryonic process in the medfly. Furthermore, a putative homologue of the *Zelda *gene has been considered, which in *D. melanogaster *encodes a DNA-binding factor responsible for the maternal-to-zygotic transition.

**Conclusions:**

Our novel sexing method facilitates the study of i) when the MTZ transition occurs in males and females of *C. capitata*, ii) when and how the maternal information of "female-development" is reprogrammed in the embryos and iii) similarities and differences in the regulation of gene expression in *C. capitata *and *D. melanogaster*. We suggest a new model for the onset of the sex determination cascade in the medfly: the maternally inherited *Cctra *transcripts in the female embryos are insufficient to produce enough active protein to inhibit the male mode of *Cctra *splicing. The slow rate of development and the inefficiency of the splicing mechanism in the pre-cellular blastoderm facilitates the male-determining factor (M) activity, which probably acts by inhibiting CcTRA protein activity.

## Background

The onset of embryonic development is the result of a complex interaction between maternal and zygotic genetic information: during the maternal-to-zygotic transition (MTZ) embryos integrate post-transcriptional regulation of maternal transcripts with transcriptional activation of the zygotic genome [1].

In the model insect *Drosophila melanogaster *maternal transcripts and proteins are deposited in the egg during oogenesis and two waves of zygotic gene transcription occur in the embryos: the first between 1 and 2 h of development and the second major burst between 2 and 3 h of development [2]. For at least one third of zygotically active genes, the expression profile is coupled to the degradation of the corresponding maternal mRNA. It has been hypothesized that the degradation of maternal mRNA could be due to the activity of Pumilio, of Smaug [3,4] and of miRNAs [5] or to the presence of a general mediator of mRNA degradation in the mRNA itself, i.e. the AU-rich element [6]. The activation of the earliest zygotic genes is linked to the presence of TAGteam sites in their promoter regions; the zinc-finger protein Zelda (*zelda*, *zld*) binds to these sites and triggers the expression of the downstream genes. TAGteam sites have been found in the promoters of genes involved in early developmental processes, including cellular blastoderm formation, sex-determination and the dorsoventral-expression pattern [2].

Although the molecular mechanisms involved in the MTZ transition during the first stages of embryogenesis are being clarified in *D. melanogaster *[4], little is known about embryogenic processes including sex determination in other insect species. This lack of knowledge does not allow us to determine whether there are features common to all embryogenic processes.

The recent publication of ESTs from medfly embryos has facilitated the investigation of embryogenesis in this species [7]. The medfly is a highly invasive agricultural pest species and belongs to the family Tephritidae that is estimated to have diverged from the family Drosophilidae between 80 and 100 mya [8,9]. Even though the medfly genome is more than three times larger than that of *D. melanogaster*, a recent analysis has shown that functionally non-coding sequences are conserved between these two species and that homologous genes, such as the *even-skipped *gene (*eve*), have the same expression profile in both species [10]. Thus, it is reasonable to hypothesize that medfly embryogenesis may follow a similar sequence of events as in *D. melanogaster*. If that is the case, *C. capitata *could be used as a reference species to study the molecular mechanisms that trigger embryogenesis, as a big genome facilitates the identification of *cis*-regulator elements through comparative genomic methods [10].

It is known that in the medfly the cellular blastoderm formation occurs between 9 and 11 h after oviposition [11] and one of the major events during early embryogenesis, the molecular mechanism that controls sex-determination, is well studied in *C. capitata *[12]. In order to detect sex-specific differences in the timing of gene expression and/or mRNA splicing in early developmental stages in the medfly, a primary requirement is the identification of the sex of the early life stages. Male and female embryos are not morphologically distinguishable [13]; however the sexes may be recognized in the early life stages using PCR-based approaches. These sexing assays are based on the repetitive nature of the medfly Y chromosome. Several repetitive DNA sequences have been isolated in medfly that are Y-specific or enriched on the Y chromosome [14]. Copies of these repetitive sequences, which appear to occur in tandemly linked arrays, are distributed over 90% of the length of the long arm of the Y chromosome [15]. The basic structure of the repeats is similar: each unit (1.3-1.7 kb) contains an AT-rich (83-87%) internal region (200 bp or more), called the A-T element. Although three previous approaches [16-18], based on the repetitive nature of the Y chromosome, may be suitable for determining the sex of embryos, an improved method has been developed and is reported in this paper. Using this molecular sexing approach it is possible to study the sex-specific expression of genes in early embryos; we have addressed four principal points: i) when the MTZ transition occurs in *C. capitata*; ii) when the sexual fate is established at the molecular level; iii) how the maternal information of "female-development" is reprogrammed in the embryos; and iv) how similar or different is the regulation of gene expression in early embryogenesis between *C. capitata *and *D. melanogaster*.

## Methods

### Flies

Individual samples of *C. capitata *from five laboratory strains and one natural population were considered in this study. The main laboratory strain used in this work was the ISPRA strain, established in 1968 at the European Community Joint Research Centre (Ispra, Italy), with wild flies from Sicily and Greece. The strain has been maintained in the quarantine facility at the Dept. of Animal Biology, University of Pavia (Italy) since 1979. Other laboratory strains used, maintained in the same insectary, were: Egypt II, obtained from the FAO/IAEA Agriculture and Biotechnology Laboratory (Seibersdorf, Vienna, Austria); Guatemala, obtained in 1990 from MOSCAMED (Guatemala); Israel and Hawaii established from flies collected in the corresponding countries, maintained in our laboratory since 1990 and 1992, respectively. In addition, a sample of wild flies collected in May 2007 from coffee drupes in Ruiru near Nairobi, Kenya, part of the ancestral home range of the species, was used in the study.

### Egg and embryo collection

To obtain fertilized and unfertilized eggs, two cages were set up using 200 mated or virgin ISPRA females, respectively. When the flies were five-days old, eggs were collected over a 5 min period. The eggs were maintained at 25°C and 65% humidity for set time intervals. After dechorionation (using 1.5-2% hypochlorite solution), the eggs were repeatedly washed in distilled water and individually transferred to 1.5 ml microcentrifuge tubes.

### Nucleic acid preparations

DNA and RNA were isolated from individual embryos using TRIzol^® ^Reagent (Invitrogen, Carlsbad, CA, USA) following the manufacturer's protocol. The extracted RNA was resuspended in RNAse-free water. Genomic DNA was individually extracted from 3^rd ^instar larvae, 7-day old pupae and 3-day old adult flies using the phenol-chloroform extraction method [19]. Following treatment with RNAse A, the DNA was extracted with phenol/chloroform, precipitated with ethanol and resuspended in TE (10 mM Tris-HCl, pH 8, 1 mM EDTA). The DNA concentration was quantified using a Nanodrop ND-1000 spectrophotometer (Nanodrop Technologies Inc., Wilmington, DE, USA).

### PCR amplifications and agarose gel electrophoresis

Oligonucleotide primers CcYf (5'-gctcgaagacatgcattgaa-3') and CcYr (5'-gacggtaagtgccattcgtt-3') were designed on a known Y-specific sequence of *C. capitata *[GenBank:AF115330] using Primer3 [20]. PCR amplifications were performed in 10 μl reaction volumes using ~50 ng DNA, 1.5 mM MgCl_2_, Reaction Buffer (10 mM Tris, 50 mM KCl; pH 8.3), 0.2 mM dNTPs mixture, 10 pmol of each primer and 1 unit *Taq *DNA polymerase (Invitrogen). Amplification was achieved on an Eppendorf Mastercycler Gradient using the following programme: an initial denaturing step at 94°C for 2 min; 30 cycles of denaturation at 94°C for 30 sec, annealing at 55.8°C for 1 min, extension at 72°C for 45 sec; and a final extension at 72°C for 5 min. The PCR products were analysed on 14 × 10 cm 1.5% agarose gel slabs in 1 × TAE buffer together with a 1 kb and a 100 bp ladder standard (Invitrogen). The bands were visualized by ethidium bromide staining and exposure to UV light. Bands selected for further analysis were eluted from the gel using the PureLink Quick Gel Extraction Kit (Invitrogen).

### EST bioinformatic analysis and primer design

**Figure 1:**
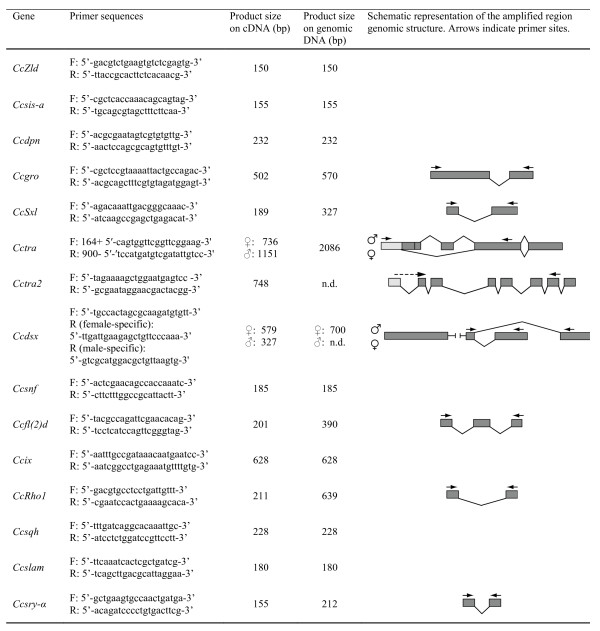
**Primer sequences, amplicon size and structure of the genomic region of the considered medfly sex-determination and cellularization genes**. Sex-specific amplicon sizes are presented where appropriate. The genomic structure is shown only for amplicons that span more than one exon and is complete for only the *Cctra *and *Cctra2 *genes.

ESTs [7] were subjected to bioinformatic analysis via BLASTX against the *D. melanogaster *genome and sequences similar to genes involved in sex determination and cellular blastoderm formation were selected (Table 1). Data on the *D. melanogaster *gene expression profiles were obtained from the flybase server [21]. The primers used to amplify gene-specific sequences by reverse transcription PCR (RT-PCR) were designed using Primer3 and may span one or more exon sequences (Figure 1).

**Table 1:** Medfly homologues of genes involved in sex-determination and cellularization and their function and embryonic expression in *D. melanogaster*

Gene, (symbol), GenBank Acc.	*Drosophila *gene, FlyBase ID	Alignment length (aa)	e-Value	Identity (%)	Similarity (%)	*D. melanogaster *gene functions	*D. melanogaster *gene expression during early embryogenesis
*CcZelda* *(CcZld)* FG073766	*Zelda(zld)* *CG12701*	138	4E-43	54	66	zinc-finger transcription factor involved in MTZ process and neuronal development	maternal origin
*Ccsisterless A* *(CcsisA)* FG075887	*sisterless-a (sisA)* *CG1641*	180	3E-13	26	49	transcription factor forming the primary signal of the sex-determination cascade (XSEs) and involved in endoderm development	expressed in female zygote at the end of nuclear cycle 8 (1:12 h)
*Ccdeadpan* *(Ccdpn)* FG076265	*deadpan (dpn)* *CG8704*	48	1E-10	75	83	transcription factor forming the primary signal of the sex-determination cascade (autosomal) and involved in dendrite morphogenesis	expressed in the zygote starting from nuclear cycle 12 (2 h)
*Ccgroucho* *(Ccgro)* FG077761	*groucho (gro)* *CG8384*	217	6E-07	97	98	corepressor of transcription forming the primary signal of the sex-determination cascade (autosomal) and involved in dendrite morphogenesis	maternal origin, *gro *transcripts are detectable until stage 11 after complete gastrulation (5:20 h)
*CcSex-lethal* *(CcSxl)* AF026145	*Sex lethal (Sxl)* *CG18350*	346	5E-113	66	73	RNA-binding protein, member of the sex-determination molecular cascade	expressed in the zygote starting from nuclear cycle 12-13 (2 h)
*Cctransformer* *(Cctra)* AF434936 AF434937 AF434938	*transformer (tra)* *CG16724*	11	4E-03	72	90	RNA-binding protein, member of the sex-determination molecular cascade	maternal origin; an increased amount of *tra *transcripts is detectable 2-3 h after oviposition
*Cctransformer2* *(Cctra2)* EU437408	*transformer2 (tra2)* *CG10128*	166	2E-42	56	72	RNA-binding protein, member of the sex-determination molecular cascade	maternal origin. No change in *tra2 *transcript amount is detectable after 2-3 h and in chromosome 2R deleted embryos
*Ccdoublesex* *(Ccdsx)* AF434935 AF435087	*doublesex* *CG11094*	336a 248b	2E-55 1E-56	68 85	77 91	zinc-finger transcription factor involved in sex differentiation process	*dsx *transcripts are first detectable during stage 11 (9:20 h) in the fat bodies and gonad primordia
*Ccsans-fille* *(Ccsnf)* FG082648	*sans fille (snf)* *CG4528*	80	2E-39	97	98	U1 snRNA binding protein involved in sex-determination and oogenesis	the SNF protein localizes to the nucleus in all tissues during development
*Ccfemale-lethal d* *(Ccfl(2)d)* FG070226 FG073068 FG071876	*female lethal d (fl(2)d* *CG6315*	493	3E-91	47	53	RNA splicing factor involved in sex-determination and female-germline sex-determination	maternal origin
*Ccintersex* *(Ccix)* FG072637	*intersex (ix)* *CG13201*	142	2E-54	71	85	transcription factor binding protein involved in sex-differentiation process	maternal origin; an increased amount of *ix *transcripts is detectable 2-3 h after oviposition
*CcRho1* *(CcRho1)* FG083938	*Rho1 (Rho1)* *CG8416*	178	5E-101	100	100	protein kinase coupled with GTPase activity involved in cellular-blastoderm formation	maternal origin
*Ccspaghetti-squash* *(Ccsqh)* FG077819	*spaghetti squash (sqh)* *CG3595*	153	1E-83	96	98	myosin heavy chain binding protein coupled with ATPase activity involved in cellularization and ovarian follicle cell development	maternal origin
*Ccslow-as-molasses* *(Ccslam)* FG068639 FJ460697	*slow as molasses (slam)* *CG9506*	836	4E-98	32	48	protein of unknown function involved in cellularization	*slam *expression rapidly increases during cycle 11 (1:45 h) and peaks during cycle 14 (2:30 h)
*Ccserendipity-α* *(Ccsry-α)* FG072156 FJ460703	*CG8247*	629	3E-91	33	55	Similar to *serendipity*-α gene product, involved in cellularization	maternal origin

^a^male specific isoform; ^b^female-specific isoform

### cDNA preparation and RT-PCR

cDNA was generated using the cloned AMV first-strand synthesis kit (Invitrogen) according to the manufacturer's instructions. The RT-PCR mixture and the cycling conditions were as reported above, but the annealing temperature was 59°C. Established primers and cycling conditions were used to amplify the *Cctra *sequences [22]. For each set of RT-PCR amplifications control reactions were performed excluding reverse transcriptase. RT-PCR amplifications were considered only when these negative controls produced no amplification products. Electrophoresis and visualization of amplification products were performed as described above.

### Cloning and Sequencing of PCR and RT-PCR Products

Gel eluted PCR and RT-PCR products were cloned into the PCR^®^2.1-TOPO^® ^vector using the TOPO TA cloning kit (Invitrogen). Positive colonies were selected and the size of the insert quantified by *Eco *RI digestion and electrophoresis. Clones were sequenced using an ABI-310 automatic sequencer and the ABI Prism BigDye Terminator Cycle Sequencing Ready Reaction Kit v. 3.1 (Applied Biosystems, Foster City, CA, USA).

### Sequence Analysis

Nucleotide sequences were aligned using T-coffee [23]. Sequence comparisons were performed using the BLAST family of programs from the National Centre for Biotechnology Information [24]. Identity values were calculated using PAUP 4.0b10 [25].

### *Southern hybridization *analyses

Genomic DNA from individual flies (4 μg) was digested with *Ssp*I endonuclease. The digested DNAs were electrophoresed on a 20 × 14 cm 1% agarose gel in 1 × TBE and transferred to a positively charged nylon membrane according to Southern [26]. The membrane was hybridized at 55°C with 200 ng of probe DNA labelled with the Gene Images Alkphos Direct labelling system (GE Healthcare, Little Chalfont, UK) using the random primer method. The hybridization and detection protocols were those described by the manufacturer. Signal detection was performed using CDP-star followed by exposure to autoradiographic film (X-OMAT AR, Kodak).

### Chromosome preparation and *in situ *hybridization

Mitotic chromosome spreads were obtained using medfly third instar larvae [27]. Briefly, brain tissue was incubated in 1% sodium citrate for 10 min at room temperature and transferred to methanol-acetic acid 3:1 solution for 4 min. The material was disrupted in 100 μl 60% acetic acid and dropped onto clean slides and dried. Pre-hybridization was performed according to [15]. *In situ *hybridization was performed using the following protocol: the probe DNA was labelled using the Biotin High Prime kit (Roche, Basel, Switzerland) and detection of hybridization signals was performed using the Vectastain ABC elite kit (Vector Laboratories, Burlingame, CA, USA) and DAB (3,3'-Diaminobenzidine tetrahydrochloride, Sigma-Aldrich, St. Louis, MO, USA) [28].

## Results and Discussion

### A putative MITE insertion on the Y chromosome permits a fast sex-assignment

The PCR-based sexing method reported here was designed to easily assign sexual identity to the earlier medfly life stages in order to study sex-specific expression and/or mRNA splicing of early, zygotically transcribed genes. This method follows previous sexing assays [16-18] that used primers to amplify a Y-specific repetitive sequence (Y114). In the previous assays, amplification was limited to the male: the presence of a sex-specific pattern only in males may give a false sex assignment should the amplification fail.

**Figure 2:**
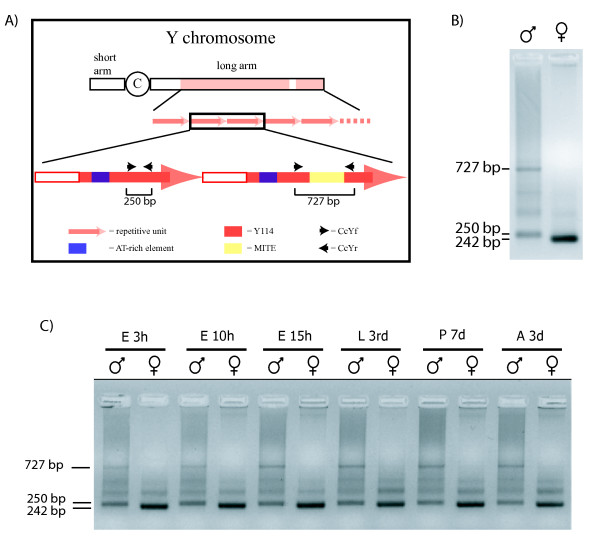
**The novel molecular sexing system in *Ceratitis capitata***. A) Schematic representation of the Y chromosome-derived repetitive sequences. Y114 (red arrows) is the reference sequence, which was used as probe in the *in situ *experiments and it was the first clone derived from these sequences [14,15]. The AT-rich region (blue boxes) is a 200 bp-long sequence characteristic of Y114. Black arrows indicate CcYf and CcYr primer positions. B) Amplification patterns of genomic DNA from individual males and females using the CcYf/r primers (see Methods). C) Developmental patterns of amplification products from genomic DNA from individual male and female *C. capitata*. E: embryos 3, 10 and 15 hr after oviposition; L 3rd: 3^rd ^instar larvae; P 7 d: 7 day old pupae; A 3 d: 3 day old adults.

The CcYf and CcYr primers were designed outside of the AT-rich element of the Y-specific sequence to amplify a 250 bp sequence (Figure 2A). Genomic DNA from individual male and female adults from the ISPRA laboratory strain was subjected to PCR amplification and, unexpectedly, amplification products were obtained in both sexes. The amplification pattern was different in males and females: only one distinct band of 242 bp was amplified in females, whereas two distinct bands of 250 bp and 727 bp were amplified from the male samples (Figure 2B). These male- and female-specific amplification patterns remain constant in all medfly laboratory strains analyzed (Egypt II, Guatemala, Israel and Hawaii) and from wild flies collected in Kenya, the home range of *C. capitata *[29], indicating that there is no pattern variability, irrespective of the geographic origin of the samples. Moreover they are constant in all developmental stages, including early embryos (Figure 2C).

**Figure 3:**
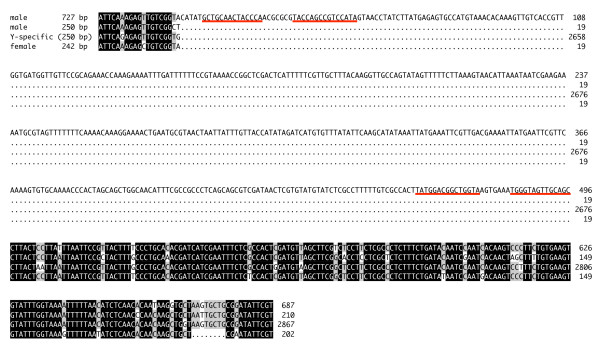
**Nucleotide alignment**. Nucleotide alignment of the male-specific 727 bp and 250 bp sequences and female-specific 242 bp sequence with the Y-specific repetitive sequence [GenBank:AF115330]. Black shading represents areas of identity within all four sequences; grey shading represents areas of identity between three sequences only. Underlined sequence represents the inverted terminal repeats of the MITE.

**Figure 4:**
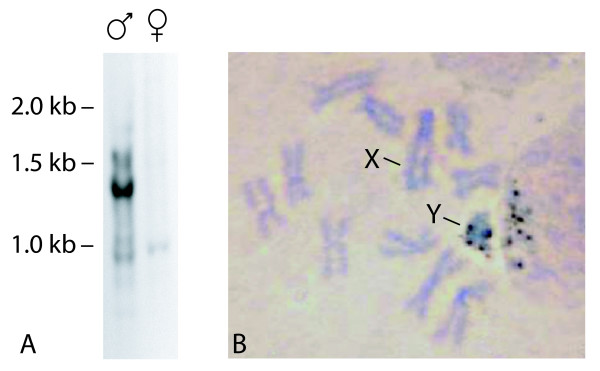
**Male-specificity of the 727 bp sequence**. A) Southern blot analysis on *SspI*-digested male and female genomic DNA using the male-specific 727 bp band as a probe. B) Localization of the sequence 727 bp on the Y chromosome by *in situ *hybridization on mitotic chromosomes.

The 242, 250 and 727 bp amplicons were sequenced [Genbank: GU122238-Genbank: GU122240] and BLASTN analyses showed that they share high identity with the Y-specific repetitive DNA sequence (ranging from 93% to 95%). Alignments among the three sequences indicated a high degree of identity (89.6 to 94.5%) (Figure 3). The male-specific 727 bp sequence, used as probe in Southern blot analysis on male and female genomic DNA, resulted in multiple intense signals with male-derived DNA and at least one weaker signal with female-derived DNA samples (Figure 4A). *In situ *hybridization on mitotic chromosomes again using the 727 bp sequence as a probe clearly indicated that this is a repetitive sequence located on the long arm of the Y chromosome (Figure 4B).

The female 242 bp sequence lacks eight base pairs in the 3' region, that are present in both male sequences, while the male 727 bp sequence contains an additional 477-bp sequence, which is absent from the other two fragments. This indel has no homology to the Y-specific repetitive DNA sequence, but it shares 86-89% identity with two medfly ESTs [7], the functional/biological roles of which are unknown. The presence of terminal inverted repeats in these sequences, their small size, the absence of coding sequence, the potential to form a stable secondary structure, the presence of duplicated target regions in the ESTs and the presence of multiple copies in the medfly genome, as confirmed by Southern hybridization (Additional file 1, Figure S1; Additional file 2, Figure S2), led us to hypothesize that the 467 bp indel sequence is a miniature inverted repeat transposable element (MITE) [30]. It is the insertion of the putative MITE into one or more repetitive units on the Y-chromosome that results in the amplification of the male-specific 727 bp sequence (Figure 2A).

### The dynamics of the sex determination molecular cascade in early embryos

The MTZ transition process integrates the post-transcriptional regulation mechanisms of the maternally inherited transcripts and the transcriptional activation and control of the zygotic genome. Particular attention has to be focused on the events that regulate the sex determination process, because the zygote has to reset the information of "female development" inherited from its mother to its own sex-development information. This process involves the activities of transcriptional and of mRNA-splicing regulators.

Three of the medfly genes involved in the molecular sex-determination cascade have previously been identified, *Cctra *[22,31], *Cctra2 *[7,32], and *Ccdsx *[33]. *Cctra *is the key-gene of the sex-determination cascade: it generates mRNAs encoding full-length active proteins only in females and displays an autocatalytic function, which guarantees the female-specific development of cell memory. *Cctra*, in cooperation with *Cctra2*, determines the sex-specific splicing of *Ccdsx*, the transcription factor that is the regulator of the sex-differentiation process.

**Figure 5:**
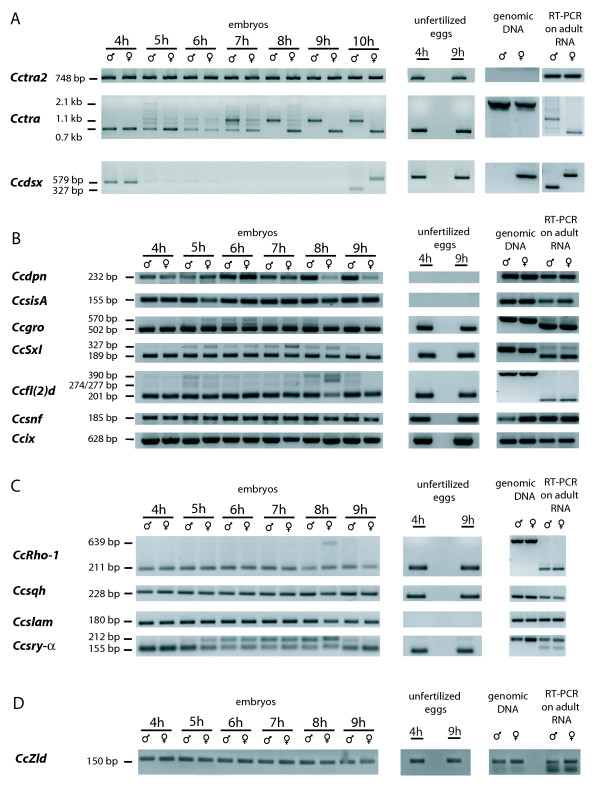
**RT-PCR expression pattern analysis during the early stages of embryogenesis**. For each of the genes analyzed by RT-PCR the expression pattern in syncitial-stage embryos is reported together with control RT-PCR amplications on cDNA derived from unfertilized eggs and adult heads. The same primers used in RT-PCR were also used in PCR amplifications on genomic DNA. A) Expression patterns of the three known *C. capitata *sex determination genes: *Cctra2, Cctra *and *Ccdsx*. For the *Cctra *gene, the 1.1 kb splicing form is the expected male-specific mRNA, while the 0.7 kb is the female-specific form. For the *Ccdsx *gene the 579 bp splicing form is the expected female-specific mRNA, while the 327 bp is the male-specific form. Absence of amplification of *Ccdsx *from adult male genomic DNA is probably due to the presence of a long intron in the male-specific amplicon (see Figure 1). B) Expression patterns of medfly genes that share similarities to known *D. melanogaster *sex determination genes. C) Expression patterns of medfly genes that share similarities to known *D. melanogaster *cellular blastoderm formation genes. D) Expression pattern of *Cczelda *gene, similar to the *D. melanogaster *key regulatory gene of the MTZ transition event.

*Cctra *and *Ccdsx *mRNAs are maternally inherited as female-specific splicing variants; the maternal information for female-specific development is reset in embryos through the reprogramming of *Cctra *mRNA splicing and the degradation of the maternal *Ccdsx *mRNAs (Figure 5A).

**Figure 6:**
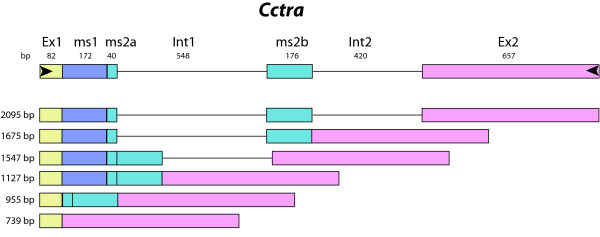
**Schematic representation of *Cctra *transcripts detected in 6 h embryos**. The colored boxes represent the exons. Male-specific exons have the prefix ms. Unspliced, spliced, and partially processed transcripts were detected in male and female embryos.

*Cctra *expression in the zygote starts between 4 and 5 h after oviposition. In 5 to 8 h embryos of both sexes, the *Cctra *mRNA population is heterogeneous, composed of transcripts of different molecular weights (Figure 5A). These patterns are not attributable to contaminating genomic DNA as controls showed that there are no amplification products in the absence of retro-transcriptase. Notably, in 6 h embryos the *Cctra *amplification patterns appear to be almost identical in both sexes. Sequencing of these transcripts showed that both male and female embryos contain six transcripts including the mature male and female *Cctra *mRNA and partially or unprocessed *Cctra *transcripts [GenBank: GU122232-Genbank: GU122237]. Moreover, the splicing mechanism does not appear to act in an orderly manner as transcripts with different combinations of intron/exon sequences are detected (Figure 6).

The presence of different splicing transcripts, including male-specific transcripts, in female embryos indicate that the maternal *Cctra *transcripts do not produce enough active CcTRA protein to prevent the male mode of splicing in female embryos. Concentration-dependant splicing activity is a general property of proteins belonging to the SR-superfamily of splicing factors [34] which includes the products of *Cctra *and *Cctra2*. Consequently, we suggest that in female embryos, the *Cctra *autoregulation loop produces more and more active CcTRA female protein, reaching the threshold concentration that results in the female-specific splicing of all the *Cctra *transcripts at 8-9 h (Figure 5A). By contrast, in male embryos the postulated Y-linked male-determining factor, M [15,22,32], directly or indirectly blocks the *Cctra *autoregulation loop, inducing the translation of more and more inactive CcTRA protein, resulting in the male-specific splicing of all the *Cctra *transcripts.

Apart from these sex-specific differences in splicing, as we cannot detect clearcut differences in *Cctra *transcription levels between male and female embryos, it is unlikely that the M factor interferes with *Cctra *transcription. Even if we cannot completely exclude this hypothesis, we presume that the M factor acts by affecting *Cctra *mRNA splicing or CcTRA protein activity (e.g. reducing its phosphorylation).

Sequencing of the *Cctra *transcripts from embryos also permitted the characterization of the genomic organization of the gene: the exon/intron structure is different from that reported previously [31]. In particular, we found that intron 1 is 50 bp longer than expected and we found no evidence of the presence of a third 52 bp long intron within the genomic portion that we analyzed. This discrepancy could be due to the general genetic variability that occurs between populations of different geographical origins and between different laboratory strains.

In contrast to *Cctra*, the *Cctra2 *transcripts appeared in RT-PCR experiments as a single band in both sexes at all the embryonic stages analysed, even though the primers spanned five introns (Figure 1). The presence of only the mature transcript, as ascertained by comparison with the published genomic sequence [Genbank:EU437408], could be due to *Cctra2 *not being expressed in the zygote during early embryogenesis and that only maternal *Cctra2 *mRNA supplies the required CcTRA2 protein. This hypothesis is supported by the observation in *D. melanogaster *that the amount of *tra2 *transcripts is lower in 2-3 h than in 0-1 h embryos, suggesting that there is no zygotic expression or that the zygotic expression cannot compensate for the concomitant degradation of the maternal mRNAs. Furthermore, in embryos in which the *tra2*-carrying chromosome 2 is deleted, the quantity of *tra2 *mRNAs is similar to that in normal embryos, indicating that the maternal *tra2 *mRNA is stable and that there is no zygotic contribution [6].

As far as it concerns the third medfly sex determination gene, *Ccdsx *expression begins in 10 h male and female embryos and its transcripts are sex-specifically spliced, as by this stage only female embryos express the functional female-specific CcTRA protein (Figure 5A).

In *D. melanogaster *sex determination occurs before cellularization as it is coupled with dosage compensation [35]. Our data show that sex determination in the medfly also occurs before cellularization. This is surprising as the medfly X chromosome is largely heterochromatic and contains very few genes, suggesting that there is no need for dosage compensation [35]. In both species, the establishment of this cascade during the first stages of embryogenesis requires the activity of proteins that act in a concentration-dependent manner. These molecular events could be facilitated in a syncitial stage rather than in a cellularized organism.

Our results show that the medfly sex determination cascade is complete at 10 h, before the end of cellularization (9-11 h [11]), whereas in *D. melanogaster *it ends after gastrulation (stage 13) [36]. A possible explanation for this phenomenon may be the shorter cascade in *C. capitata *and also the late onset of cellularization (9-11 h) compared to 2.10-2.45 h in *D. melanogaster*.

### Possible medfly sex determination-related genes

In *D. melanogaster *the ratio of X-linked and autosomal gene products represents the primary signal of the sex determination cascade. Among these genes, *sisterless A *(*sisA*) is one of the X-linked genes and *deadpan *(*dpn*) and *groucho *(*gro*) are autosomal genes. Early during development, the ratio of their product concentrations, together with those of other genes, determines the expression status of the key gene *Sex-lethal *(*Sxl*) starting from an early promoter (*Sxl*^Pe^) [37,38]. In females the *Sxl *early mRNA encodes an active SXL protein. At later developmental stages a maintenance promoter (*Sxl*^Pm^) triggers *Sxl *transcription in both males and females, but this pre-mRNA requires the presence of an active SXL protein before it can mature into full-length SXL-encoding mRNA. Only in female embryos does the SXL translated from *Sxl*^Pe ^mRNA act as the memory device for female sexual development via its auto-regulatory function. The function of SXL requires the activity of cofactors, such as *sans-fille *(*snf*) and *female lethal (d) *(*fl(2)d)*. When *Sxl *is activated, it sets in motion a cascade of regulatory genes, *transformer (tra)*, *transformer-2 (tra2) *and *doublesex *(*dsx*) [39]. In female embryos the DSX^F ^protein cooperates with the product of the *intersex *(*ix*) gene to drive the expression of the genes responsible for sexual differentiation [40].

Even though the *C. capitata *sex determination cascade differs from that of *D. melanogaster*, several medfly EST transcripts with homology to the *sisA*, *dpn *and *gro *genes were identified (Table 1). Their expression in the embryos comply with the classification of their *D. melanogaster *homologues: *CcsisA *and *Ccdpn *are expressed by the zygote earlier than 4 h after oviposition while *Ccgro *is a maternal gene, as its transcripts are inherited from the mother (Figure 5B). There were no apparent differences in expression between male and female embryos, apart from an apparent lower amount of *Ccdpn *transcripts in the 8-9 h female embryos, compared to their male counterparts. This difference has to be confirmed with further analyses and its biological significance has to be investigated. As the medfly primary sex determination signal is not based on the ratio of X and autosomes gene products, the presence of transcripts of these genes during embryonic development opens the interesting prospect of identifying the roles that they play during early medfly development.

As expected, the *CcSxl *gene has no sex-specific splicing variants and it is inherited maternally as a mature transcript. This difference with respect to *D. melanogaster *was also reported for *MdSxl *in *Musca domestica *[41], a species that appears to have a sex determination system similar to that of the medfly [42]. This is indicative of a fundamental difference in transcriptional regulation of this gene and shows that this gene has a different function in drosophilid and non-drosophilid species.

The medfly embryos also inherit transcripts of the *Ccsnf, Ccfl(2)d *and *Ccix *genes. These genes could have the same functions in the medfly as their *Drosophila *homologues, as co-regulators of *Cctra *splicing (*Ccfl(2)d *and *Ccsnf*) or as a co-factor of *Ccdsx *(*Ccix*).

Like *Cctra*, the *Ccgro*, *CcSxl *and *Ccfl(2)d *maternal genes present at least two PCR products in 5 to 8 h old embryos in both sexes (Figure 5B). Sequencing demonstrated that the larger fragment corresponds to the pre-mRNA, while the smaller fragment is the spliced/mature mRNA. This suggests that the newly transcribed mRNAs are not immediately processed into mature forms.

To conclude, we can hypothesize that two waves of zygotic expression occur in the medfly: the earliest genes, which include *CcsisA *and *Ccdpn*, are expressed before 4 h after oviposition. The second major burst of transcription occurs at 5 h after oviposition and the *Ccgro, CcSxl, Cctra *and *Ccfl(2)d *genes belong to this second wave.

### Towards the cellular blastoderm formation

A second major event during the first stage of embryogenesis is the cellular blastoderm formation, which in *C. capitata *occurs between 9 h and 11 h after oviposition [11].

In *D. melanogaster *a set of genes is involved in this process that includes maternal, maternal-zygotic and zygotic genes. In the medfly EST database we identified two sequences that are similar to the *D. melanogaster *maternal genes, *Rho1 *and *spaghetti-squash (sqh)*. These genes are also maternal in the medfly and the immature form of *CcRho1 *is present in 5-8 h old embryos, indicating that this gene is expressed during the second major wave of transcription in the embryo (Figure 5C).

The *slow as molasses (slam) and serendipity-α *(*sry-α*) genes are expressed only in the zygote in *D. melanogaster*. The *Ccslam *gene complies with this classification, whereas the *Ccsry-α *gene is maternally inherited in the embryos as a mature transcript. Again, in 5 to 8 h old embryos, the immature form of the *Ccsry-α *mRNA was detected. The recent embryonic *in situ *detection of *Ccsry-α *did not reveal the presence of maternal transcripts of this gene [43]. This discrepancy may be due to the higher sensibility of a PCR- compared to a hybridization-based approach. What is more, the *Ccsry-α *gene showed greater amino-acid similarity to the *D. melanogaster *CG8247 gene than to *sry-α*. CG8247 is a *sry-α*-like gene that is also involved in cellular blastoderm formation but is, unlike *sry-α*, inherited maternally in *D. melanogaster*

### The putative *CcZelda *gene

In *D. melanogaster *the presence of TAGteam sites in promoter-sequences is a feature of genes that are expressed early during embryogenesis [44]; these sites are bound by the zinc-finger transcription factor Zelda [2]. *zelda *(*zld*) transcripts are present in the germ line cells of the ovary, in unfertilized eggs and throughout early embryonic development, while later on in development *zld *becomes restricted to the nervous system. The Zld protein is a general activator of early genes and it is responsible for the MTZ transition. It regulates the activation of genes involved in the three early processes that occur in the embryos, sex determination (including *sisA*, *Sxl *and *dpn*), cellular blastoderm formation (including *slam *and *sry-α*), and the dorsoventral genes.

In the medfly EST database we identified a sequence that encodes for a protein with a high degree of identity with Zelda (Table 1). Like its putative homologue *zld*, *Cczld *transcripts are maternally derived and they are present throughout the syncitial period (Figure 5D). The promoter regions of two of the medfly genes putatively regulated by Zelda, *Ccslam *and *Ccsry-α*, are known, but it was not possible to identify conserved TAGteam sites between the medfly and *Drosophila *homologues of these genes. In the *D. melanogaster slam *gene the TAG sites are present only in the first intron, as we do not have the genomic sequence of *Ccslam *we cannot confirm that these sites are conserved in the medfly gene. In the *sry-α *gene the TAG sites are located in the promoter, whereas in *Ccsry-α *the promoter lacks these sites. This lack of TAGteam sequences in *Ccsry-α *could be a further demonstration that this gene has a different transcription-control molecular mechanism with respect to *sry-α *in *Drosophila*. The sequencing of the entire medfly genome, currently in progress, will provide a powerful tool to understand the molecular mechanisms of the MTZ transition process in this species.

## Conclusions

**Figure 7:**
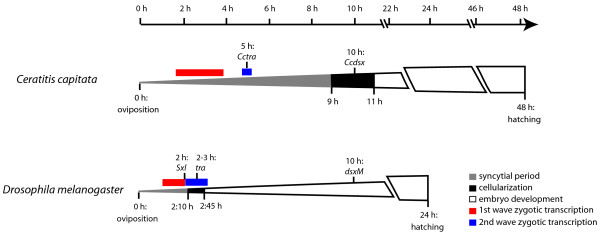
**Timing of gene expression during embryogenesis in *C. capitata *and *D. melanogaster***. A schematic representation of the medfly and *D. melanogaster *embryogenesis stages, from oviposition (0 h) to hatching (48 h in the medfly, 24 h in *D. melanogaster*). The zygotic expression onset of crucial genes of the sex determination cascade and the two zygotic expression waves are reported in both species.

The novel PCR-based sexing method reported in this paper takes advantage of a putative MITE insertion on the medfly Y chromosome and it allows the analysis of the MTZ transition at very early stages of embryogenesis in both sexes. During this period, as in *D. melanogaster*, two main events take place: sex determination and the cellular blastoderm formation. The zygotic transcriptional activation of genes involved in these processes follows two waves (Figure 7). The first wave starts before 4 h after oviposition and includes the zygotic genes *CcsisA*, *Ccdpn *and *Ccslam*. The second major burst of expression activation begins 5 h after oviposition and includes the maternal-zygotic genes *Ccgro*, *CcSxl*, *Cctra*, *Ccfl(2)d*, *CcRho1 *and *Ccsry-α*. The *C. capitata *sex determination cascade is shorter than that of *D. melanogaster*, probably due to the fact that *Cctra*, instead of *CcSxl*, is the master switch gene. Further differences in *C. capitata*, compared to *Drosophila *early development, concern the late onset of cellularization and the accomplishment of sex determination before the cellular blastoderm formation. This last issue proves that the transcription of *Ccdsx *begins during the cellularization process, while that of *D. melanogaster *occurs after the cellular blastoderm formation.

The maternal information of female development is reset in the medfly embryos through the reprogramming of *Cctra *mRNA splicing and the degradation of the maternal *Ccdsx *mRNA. The heterogeneity of the *Cctra *mRNA population during the "splicing-resetting" phase (5-8 h after oviposition) is indicative of a threshold-dependent activity of the CcTRA protein. Thus we suggest that the maternally-inherited *Cctra *transcripts in female embryos are not sufficient to produce enough active protein to inhibit the male mode of *Cctra *splicing. These data suggest that the M-factor [15] acts by inhibiting the CcTRA protein activity, so that it does not reach the threshold concentration required for female-specific splicing of all the *Cctra *transcripts. The slow rate of development and the apparent inefficacy of the splicing mechanism in the pre-cellular blastoderm, exemplified by the presence of immature mRNA precursors of many genes (*Ccgro*, *CcSxl*, *Ccfl(2)d, CcRho1 *and *Ccsry-α*), could facilitate the M-factor activity. This model of sex determination in the medfly differs from that previously proposed [12].

Operationally, the slow rate of early development in the medfly may facilitate the study of the expression and splicing mechanisms that occur in slow motion compared to *D. melanogaster*, thus making *C. capitata *a suitable candidate to deepen our knowledge of the molecular mechanisms involved in the MTZ transition in Diptera.

As a practical consequence, promoter and enhancer sequences that are active in early stages of development will become available as tools for population control strategies against pest insects.

## Authors' contributions

PG, ARM and GG conceived the study, and participated in its design and coordination. PG and AF designed and performed the sex discrimination method, PCR analyses and sequencing. PS, LMG, and FS performed RNA extractions and cDNA synthesis. AZ and GF collaborated in cytogenetic analyses. PG, LMG, GG and ARM drafted the manuscript. All authors read and approved the final manuscript.

## Supplementary Material

Additional file 1 **A new MITE in *Ceratitis capitata***. A) Southern blot analysis of male and female ISPRA genomic DNA (*SspI *digested) using the indel derived from the Y727 bp sequence as probe. The multiple signals are easily visible in both male and female DNA, even if the hybridization pattern is clearly different. B) Putative secondary structure of the MITE-RNA (*in silico *analysis using RNA-fold web server). Click here for file

Additional file 2 **Alignment of the Y-derived sequence of the putative new MITE, and two sequences from the medfly EST database**. Grey boxes indicate nucleotide identities between the three sequences; red and light-blue boxes highlight the positions of the putative ITR (inverted terminal repeats), and the green boxes highlight the direct duplication of the genomic sequence of one of the three sequences. Click here for file
