# Supplementary figures and images for: Sex and the single embryo: early deveopment in the Mediterranean fruit fly, Ceratitis capitata
Source: BMC Dev Biol. 2010 Jan 26;10:12. doi: 10.1186/1471-213X-10-12 (PMC2826288; doi:10.1186/1471-213X-10-12)

A)

♂ ♀

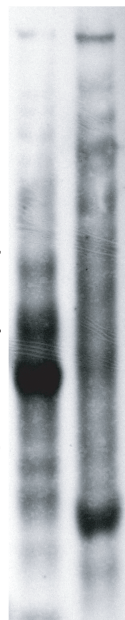

B)

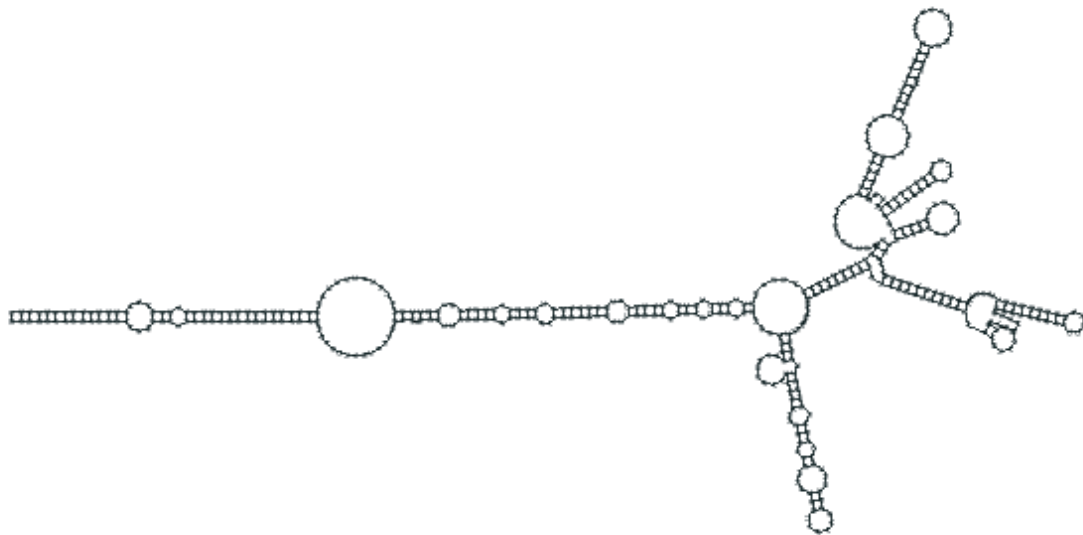

Supplement: Additional file 1 — A new MITE in Ceratitis capitata. A) Southern blot analysis of male and female ISPRA genomic DNA (SspI digested) using the indel derived from the Y727 bp sequence as probe. The multiple signals are easily visible in both male and female DNA, even if the hybridization pattern is clearly different. B) Putative secondary structure of the MITE-RNA (in silico analysis using RNA-fold web server). [file 1471-213X-10-12-S1.PDF]
