# Supplementary material for: Sex and the single embryo: early deveopment in the Mediterranean fruit fly, Ceratitis capitata
Source: BMC Dev Biol. 2010 Jan 26;10:12. doi: 10.1186/1471-213X-10-12 (PMC2826288; doi:10.1186/1471-213X-10-12)
Supplement: Additional file 2 — Alignment of the Y-derived sequence of the putative new MITE, and two sequences from the medfly EST database. Grey boxes indicate nucleotide identities between the three sequences; red and light-blue boxes highlight the positions of the putative ITR (inverted terminal repeats), and the green boxes highlight the direct duplication of the genomic sequence of one of the three sequences. [file 1471-213X-10-12-S2.DOC]

1 50

MITE (1) ----------------------------------------------GCTG

FG088299 (1) ---------------------------TAAATGGGCGACGCGTAAGGCTG

FG083766 (1) CTAACTCTAACCCTTTTTCATTATCTCTAAAGTACTCNTGACTAAGGCTG

51 100

MITE (5) CAACTACCCAACGCGCGTACCAGCCGTCCATAGTAACCTATCTTATGAGA

FG088299 (24) CAACTACCCAACGAGTGTACCAGCCGTCCATAGTAACCTATCTTATGAGA

FG083766 (51) CAACTACCCAACGAGCGTACCAGCCGTCCATAGTAACCTATCTTATGAGA

101 150

MITE (55) GTGCCATGTAAACACAAAGTTGTCACCG-------------TTGGTGATG

FG088299 (74) GCGCCATGTAAACACAAAGTTGTCACCGATTTACCATAGCGTTGCTGACG

FG083766 (101) GCACCATGTAAACACAAAGTTGTCACCGATTTACCATAGCGTTGCTGACG

151 200

MITE (92) GTTGTTCCGCAGAAACCAAAGAAAATTTGATTTTTTCCGTAAAACCGGCT

FG088299 (124) TTTGTGTCGCAGAAACCAACGAAGTTTTGATTTTT-CCGTAAAACCGGCT

FG083766 (151) TTTGTGTCGCAGAAACCAACGAAGTTTTGATTTTT-CCGTAAAACCGGCT

201 250

MITE (142) CGACTCATTTTTCGTTGCTTTACAAGGTTGCCAGTATAGTTTTTCTTAAA

FG088299 (173) CGGCTCATTTTTCGTTGCTATACAAGGTTGCCAGTATAGCCTTTCTTAAA

FG083766 (200) CGGCTCATTTTTCGTTGCTATACAAGGTTGCCAGTATAGCCTTTCTTAAA

251 300

MITE (192) GTAACATTAAATAATCGAAGAAAATGCGTAGTTTTTTTCAAAACAAAGGA

FG088299 (223) GTAACATTAAATAATCGAAAATAATGCGTTGTTTTGTTCAACAGAAATGA

FG083766 (250) GTAACATTAAATAATCGAAAATAATGCGTAGTTTTGTTCAAAAGAAATGA

301 350

MITE (242) AAACTGAATGCGTAACTAATTATTTGTTACCATATAGAT----CATGT--

FG088299 (273) AAAATAAATGCGTAACTAATTATTTGTTACCATATAGATAATACATTTTT

FG083766 (300) AAAATAAATGCGTAACTAATTATTTGTTACCATATAGATTATGTATATTT

351 400

MITE (286) -------GTTTATATTCAAGCATATAAATTATGAAATTCGTTGACGAAAA

FG088299 (323) GATTTTCGTTAATATCCGAACACATAAATTATGAAATTTGTTGACGAAAA

FG083766 (350) GCCTTGTGTTTATGTTAAAGCAAATAAATTATGAAATTCGTTGACGAAAA

401 450

MITE (329) TTATGAATTCGTTCAAAAGTGTGCAAAACCCACTAGCAGCTGGCAACATT

FG088299 (373) TTATGAATTTGTTTAAAAGTGTGCAAA-CACACAGGCAGCTGGCAACATT

FG083766 (400) TTATGAATTTGTTCAAAAGTGTGCAAA-CACACAGGCAGCTGGCAACATT

451 500

MITE (379) TCGCCGCCCTCAGCAGCGTCGATAACTCGTGTATGTATCTCGCCTTTT-T

FG088299 (422) TCGCCGCTCTCAGCGGCGTCGATAGCTCGTGTATGTATTTCACTACTGAT

FG083766 (449) TCGCCGCTCTCAGCGGCGTCGATAGCTCGTGTATGTATTTCACTACTAAC

501 550

MITE (428) GTCGCCACTTATGGACGGCTGGTAAGTGAAATGGGTAGTTGCAGC-----

FG088299 (472) GTCGTCAGTTATGGACGGCTGGTGAGT-AAATG-----------------

FG083766 (499) GTCGTCAGTTATGGACGGCTGGAGAGTGAGTTGGGTAGTTGCAGCCTAAG

551 600

MITE (473) --------------------------------------------------

FG088299 (504) --------------------------------------------------

FG083766 (549) CATATCAATATGGGAAGTTTGTATATTTCTGCTGAATTATCATTTAGATT

601

MITE (473) ---

FG088299 (504) ---

FG083766 (599) TTG
